# Overexpression of the Hsa21 Transcription Factor RUNX1 Modulates the Extracellular Matrix in Trisomy 21 Cells

**DOI:** 10.3389/fgene.2022.824922

**Published:** 2022-03-10

**Authors:** Nunzia Mollo, Miriam Aurilia, Roberta Scognamiglio, Lucrezia Zerillo, Rita Cicatiello, Ferdinando Bonfiglio, Pasqualina Pagano, Simona Paladino, Anna Conti, Lucio Nitsch, Antonella Izzo

**Affiliations:** ^1^ Department of Molecular Medicine and Medical Biotechnology, University of Naples Federico II, Naples, Italy; ^2^ CEINGE-Advanced Biotechnologies, Naples, Italy; ^3^ Department of Chemical, Materials and Production Engineering, University of Naples Federico II, Naples, Italy; ^4^ Institute of Experimental Endocrinology and Oncology “G. Salvatore”, National Research Council, Naples, Italy

**Keywords:** down syndrome, trisomy 21, chromosome 21, extracellular matrix, RUNX1, cell migration

## Abstract

Down syndrome is a neurodevelopmental disorder frequently characterized by other developmental defects, such as congenital heart disease. Analysis of gene expression profiles of hearts from trisomic fetuses have shown upregulation of extracellular matrix (ECM) genes. The aim of this work was to identify genes on chromosome 21 potentially responsible for the upregulation of ECM genes and to pinpoint any functional consequences of this upregulation. By gene set enrichment analysis of public data sets, we identified the transcription factor RUNX1, which maps to chromosome 21, as a possible candidate for regulation of ECM genes. We assessed that approximately 80% of ECM genes overexpressed in trisomic hearts have consensus sequences for RUNX1 in their promoters. We found that in human fetal fibroblasts with chromosome 21 trisomy there is increased expression of both *RUNX1* and several ECM genes, whether located on chromosome 21 or not. SiRNA silencing of *RUNX1* reduced the expression of 11 of the 14 ECM genes analyzed. In addition, collagen IV, an ECM protein secreted in high concentrations in the culture media of trisomic fibroblasts, was modulated by *RUNX1* silencing. Attenuated expression of *RUNX1* increased the migratory capacity of trisomic fibroblasts, which are characterized by a reduced migratory capacity compared to euploid controls.

## Introduction

The extracellular matrix (ECM) plays a critical structural and functional role in tissue organization and remodeling, and in the regulation of cellular processes. It is made up of collagens, proteoglycans and glycosaminoglycans, elastin and elastic fibers, laminins, fibronectins, and other proteins/glycoproteins such as matricellular proteins (Theocharis et al., 2014). The ECM guides tissue morphogenesis, development, and homeostasis, through the regulation of cellular physiology, growth, survival, differentiation, and adhesion (Karamanos et al., 2021). The ECM and its components play an essential role during embryonic development, by regulating cellular differentiation and organogenesis, as well as in adult life (Bonnans et al., 2014). The ECM regulates several aspects during neural development, such as cell shape, proliferation, differentiation and migration, in particular in developing neural tube, and neocortex (Long and Huttner, 2019). Alterations in the composition and/or organization of the ECM are linked to pathological conditions. The mechanism of just about every known disease can be traced back to some part of the matrix, typically in the form of an abnormal amount or activity level of a particular matrix component (Iozzo and Gubbiotti, 2018).

Down syndrome (DS) is a developmental disorder with multiorgan involvement (Lagan et al., 2020). It is due to trisomy of chromosome 21 (Hsa21), which causes the dysregulation of many genes on all chromosomes. It has been proposed that the presence of an extra copy of Hsa21 causes global upregulation of Hsa21 genes (primary effect) and that upregulated Hsa21 genes are responsible for the dysregulation of genes on all chromosomes (secondary effect) (Epstein, 1988; FitzPatrick, 2005; Mao et al., 2005). A significant dysregulation of genes coding for ECM components has been reported (Conti et al., 2007; Vilardell et al., 2011). It is conceivable that the consequences of this dysregulation on ECM composition and assembly may contribute to congenital defects, such as cardiac defects. Indeed, it has been proposed that the altered expression of some components of the ECM might contribute to congenital heart defects (CHD) (Gittenberger-De Groot et al., 2003), which are present in DS with a frequency of about 50% (Stoll et al., 2015). Interestingly, CHD in DS are mainly represented by endocardial cushion defects. Endocardial cushions consist of an ECM jelly, containing highly proliferative valve progenitor cells and undergoing remodeling of its components, which is necessary for subsequent morphogenetic events (Shelton and Yutzey, 2007). Also Hirschsprung’s disease (HSCR), frequently observed in DS, is thought to be caused by excess collagen deposition preventing the precursor cells of the enteric nervous system from colonizing the distal intestine (Heuckeroth, 2015).

To understand the molecular mechanisms leading to CHD in DS the transcriptional profile of human fetal hearts from DS and euploid fetuses was determined and it was found that genes coding for ECM proteins are the most over-represented among the upregulated ones (Conti et al., 2007). Here we asked the question of which Hsa21 gene(s) might be responsible for the dysregulation of ECM genes in DS and looked for evidence that the ECM dysregulation that occurs in DS has relevant functional consequences.

## Methods

### Gene Set Enrichment Analysis of Public Expression Data

A set of expression data from GSE19836 series (De Cegli et al., 2010) was obtained from the Gene Expression Omnibus repository GEO (http://www.ncbi.nlm.nih.gov/geo). This set of data was derived from the analysis of a mouse ESC bank in which 32 orthologs of human chromosome 21 genes, including transcription factors and protein kinases, were individually overexpressed in an inducible manner. A set of clones were transcriptionally profiled under inducing and non-inducing conditions with Affymetrix Gene Chip Mouse 430_2. Gene set enrichment analysis of differentially expressed genes was performed using GeneSpring software as previously described (Izzo et al., 2014), focusing on Cellular component Gene Ontology (GO) ECM categories and pathways.

Four datasets of gene expression (SET1–SET4) derived from either upregulation or downregulation of *RUNX1* expression were obtained from published data and analyzed using the Web-based Gene Set Analysis Toolkit (http://www.webgestalt.org/) (Liao et al., 2019). SET1 included expression data derived from Runx1 deficiency in mouse embryos (E12) (Michaud et al., 2008); SET2 included expression data from RNA-seq analysis after suppression of *RUNX1* expression in the human MCF-7 breast cancer cell line (Barutcu et al., 2016); SET3 included expression data from transcriptome analysis of mouse fibroblasts after *Runx1* overexpression (Wotton et al., 2008); SET4 included data from the RNA-seq transcriptome profiling of murine *Runx1* ^*KO*^ hemogenic endothelial cells (Lie-A-Ling et al., 2014). For each dataset, we submitted the list of genes positively correlated with *RUNX1* expression, selecting a reference set that was the genome, if the list of differentially expressed genes derives from an RNA-seq experiment, or the portion of genome represented on the platform (for instance array) that generated the data to be analyzed. In each dataset, we explored any enrichment of ECM categories in search of those consistently dysregulated across these studies.

### Transcription Factor Binding Site Analysis

The promoter regions from −1000bp to +0bp of mouse genes belonging to Cellular component GO ECM category and ECM genes upregulated in DS fetal heart tissues were analyzed by PSCAN software (http://159.149.109.9/pscan/) in order to detect DNA binding motifs of mouse and human RUNX1 matrix respectively [obtained from the TRANSFAC database (Matys et al., 2006)]. Genes were ranked according to their score (from 0 to 1), which gives an immediate indication of which genes are more likely to be effective targets of RUNX1. We selected genes with a positive z-score and an affinity score cut-off > 0.8.

### Ethics Statement

Human fetal fibroblasts were obtained from the “Telethon Bank of Fetal Biological Samples” at the University of Naples Federico II. All protocols related to the Bank were approved by the “Ethics Committee University Federico II”. Eight samples were from skin biopsies explanted from human fetuses with trisomy of Hsa21 (DS-HFF) and four from skin biopsies of euploid fetuses (N-HFF) after therapeutic abortion at 18–22 gestational weeks.

### Cell Culture

Fibroblasts were cultured in T25 flasks (Corning Incorporated - Life Sciences, New York, United States) with DMEM + 10% FBS (Merck, Darmstadt, Germany) supplemented with 1% penicillin/streptomycin (Gibco^™^, Thermo Fisher Scientific, Waltham, MA, United States) at 37°C in a 5% CO_2_ atmosphere. Karyotype analysis was periodically performed according to standard methods (Longobardi et al., 2021).

### RNA Extraction and Quantitative RT-PCR

RNA extraction and quantitative RT-PCR (qRT-PCR) were performed as previously described (Mollo et al., 2021). Primer pairs (MWG Biotech, Ebersberg, Germany) were designed using Primer 3 software (https://bioinfo.ut.ee/primer3/; last date accessed 01/10/2021) to obtain amplicons ranging from 100 to 150 bp. Primer efficiency was tested by generating standard curves for each gene. QRT-PCR results are presented as relative mRNA levels normalized against reference control values. Expression values were normalized either versus scrambled transfected cells or versus euploid cells. The *GAPDH* housekeeping gene was chosen as a reference gene.

### Western Blotting

Cells were lysed by using NP-40 lysis buffer (NaCl 120 mM, Tris 20 mM pH 7.5; NP-40 2%) containing protease inhibitors and nuclei were pelleted by centrifugation at 13000 rpm for 30 min at 4°C. Equal amounts of protein extracts were run on SDS-PAGE (sodium dodecyl sulphate-polyacrylamide gel electrophoresis) and transferred onto nitrocellulose membranes that were incubated with the following specific primary antibodies: rabbit polyclonal anti-collagen IV (600-401-106; Rockland Immunochemicals, Limerick, PA, United States), rabbit polyclonal anti-RUNX1 (#4334; Cell Signaling Technology, Danvers, MA, United States). Primary antibodies were revealed with HRP-conjugated secondary antibodies (GE-Healthcare, Buckinghamshire, United Kingdom) and revealed by chemiluminescence (Pierce^™^, Thermo Fisher Scientific, Waltham, MA, United States).

### Secretion Assays

Evaluation of collagen IV secretion was performed as previously described (Amodio et al., 2019). Briefly, cells grown on 60 mm dishes were incubated in culture medium containing 0.75% serum for 8 h. Equal volumes of corresponding culture mediums were then collected, TCA-precipitated, separated by 7.5% SDS-PAGE and revealed by western blotting using an anti-collagen IV antibody.

### Transfection Protocol

*RUNX1* was transiently silenced in five DS-HFF lines using Interferin (Polyplus transfection, Illkirch, France) as transfection reagent and a pool of specific *RUNX1*-siRNAs (ON-TARGETplus SMARTpool, Dharmacon^™^, Horizon Discovery, Waterbeach, United Kingdom). A pool of non-targeting siRNAs (ON-TARGETplus SMARTpool Non-targeting siRNAs control, Dharmacon^™^) was used as negative control (SCR). Cells were plated on 100 mm dishes (Corning Incorporated - Life Sciences) (300,000 cells/well) for RNA and protein collection, and on 60 mm dishes for the scratch assay. DS-HFFs were transfected with 20 nM siRNA according to the manufacturer’s protocol (Polyplus transfection). Seventy-two hours after transfection, the effects of siRNA-mediated *RUNX1* attenuation were evaluated.

### Wound Healing Assay

Fibroblasts were seeded on 60 mm culture dishes and, once 90% confluency was reached (generally 72 h for each experimental condition), a “wound” was made by scratching the cell monolayer in a straight line with a sterile P-200 pipette tip, in the center of the dish to only detach central cells. Dishes were washed twice with 1 ml of sterile 1X PBS to remove the floating cells and debris and 2 ml of serum-free medium was added for the assay. Images were acquired by microscopy at 0 h and at the desired time points (24 and 48 h). Image analysis was performed using ImageJ software, version 1.52t (Fiji) to quantify and compare wound closure across different conditions.

### Statistical Procedures

All assays were performed independently and in triplicate, unless otherwise indicated. Statistical analysis was performed using GraphPad Prism software version 5.0 (GraphPad Software, La Jolla, CA, United States; http://www.graphpad.com). Student’s *t*-test was applied to evaluate the statistical significance of differences measured throughout the data sets presented. The threshold for statistical significance (*p*-value) was set at 0.05.

## Results

### Analysis of Public Expression Data Suggests That RUNX1 Affects ECM Gene Expression

Genes coding for ECM proteins are globally overexpressed in trisomic fetal hearts (GEO GSE1789) (Conti et al., 2007). In light of this result, we investigated whether one or more Hsa21 genes, whose expression is upregulated as a consequence of trisomy, might be responsible for the altered expression of ECM genes. The re-analysis of mouse gene expression data obtained after over-expression of individual Hsa21 genes (GEO GSE19836) (De Cegli et al., 2010), showed that the transcription factor Runx1 is able to induce the upregulation of ECM genes. Indeed, 24 h after induction, *Runx1* was about 3 times overexpressed causing the upregulation of 534 genes (logFC > 0.3, adjusted *p*-value < 0.01). “Extracellular matrix” (GO:0031012) was the most enriched Cellular Component GO category (*p*-value = 4.2e−6) (Table 1), with a cluster of 37 upregulated genes (Supplementary Table S1). No other Hsa21 transcription factor or regulator analyzed was able to induce similar effects. This result suggests that ECM genes may represent a specific target of RUNX1.

**TABLE 1:** Gene Ontology categories affected by *Runx1* overexpression in GSE19836 dataset.

Gene set	Description	Observed upregulated genes	Enrichment ratio	Adjusted *p*-value
GO:0015629	Actin cytoskeleton	38/427	2.25	1.9e−6
GO:0031012	Extracellular matrix	37/424	2.21	4.2e−6
GO:0005911	Cell-cell junction	35/436	2.03	4.6e−5
GO:0098589	Membrane region	32/385	2.10	5.1e−5
GO:0043230	Extracellular organelle	12/88	3.45	1.6e−4
GO:0031252	Cell leading edge	31/397	1.97	2.1e−4
GO:0005769	Early endosome	24/309	1.96	1.1e−3
GO:0000323	Lytic vacuole	29/404	1.81	1.3e−3
GO:0001891	Phagocytic cup	5/23	5.50	1.7e−3
GO:0098858	Actin-based cell projection	16/207	1.95	7.8e−3

Upregulated genes after *Runx1* overexpression were analyzed by Webgestalt software for GSEA in Cellular component categories. The table indicates for each GO category: the number of observed genes out of all genes belonging to such category represented on the platform used for the analysis; the enrichment ratio of observed to expected genes; the adjusted *p*-value.

### Modulation of *RUNX1* Dysregulates the Expression of ECM Genes

To further support the notion that RUNX1 may regulate ECM gene expression, we performed gene set enrichment analysis (GSEA) on four datasets of gene expression (SET1–SET4; see Methods), from experiments in which *RUNX1* gene had been either upregulated or downregulated. These analyses showed that the ECM was consistently among the most affected Cellular Component GO categories in different organisms and conditions (Table 2; Supplementary Table S2). Other relevant categories affected by *RUNX1* dysregulation were “Cell-substrate junction” (GO:0030055), “Collagen trimer” (GO:0005581), and “Protein complex involved in cell adhesion” (GO:0098636). Intersection among the four datasets did not show an extensive overlap (Supplementary Table S3). Comparison between the list of ECM genes upregulated in DS fetal hearts (Conti et al., 2007) and the list of ECM genes downregulated after *Runx1* modulation demonstrates the overlapping of seven genes with SET1 (Michaud et al., 2008), including *ADAMTS5*, *COL5A1*, *COL18A1*, and *FBLN1*, and eight genes with SET2 (Barutcu et al., 2016), including *COL5A1*, *FBLN1*, and *MMP2*. This approach helped us to prioritize genes for further studies on RUNX1 targets. The above analyses support the hypothesis that RUNX1 is involved in the regulation of ECM genes.

**TABLE 2:** Enrichment ratio of “Extracellular matrix” GO category for each dataset analyzed.

Dataset	*RUNX1* modulation	Observed dysregulated ECM genes	Enrichment ratio	Adjusted *p*-value
SET1 Michaud et al. (2008)	Deficiency in mouse embryos	72/424	1.51	1.9e−4
SET2 Barutcu et al. (2016)	KO in human MCF-7	28/496	2.77	8.2e−7
SET3 Wotton et al. (2008)	Overexpression in mouse fibroblasts	10/424	3.72	2.8e−4
SET4 Lie-A-Ling et al. (2014)	KO in hemogenic endothelium murine cells	21/441	3.83	9.4e−4

Dysregulated genes after *RUNX1* modulation were analyzed by Webgestalt software for GSEA in Cellular component categories. The table indicates for each dataset: the type of modulation; the number of observed genes out of all genes belonging to ECM category represented on the platform used for the analysis; the enrichment ratio of observed to expected genes; the adjusted *p*-value.

### ECM Genes Show Consensus Sequences to RUNX1 in Their Promoters

The results of bioinformatics analyses, which suggest a role of RUNX1 in the control of ECM gene expression, prompted us to ask whether the promoters of ECM genes contain binding sites for the transcription factor RUNX1. To this end, we used PSCAN software (Zambelli et al., 2009) to analyze the presence of consensus sequences to the “*Position Frequency matrix*” of RUNX1. We first analyzed the promoter region from −1,000 to +0, relative to the Transcription Starting Site, of each of the genes belonging to the mouse GO ECM category. We found significant affinity of the mouse RUNX1 matrix for the set of promoters of the analyzed genes (*p*-value = 5e−3) (Supplementary Figure S1). Overall 293 of the 534 analyzed genes, i.e., approximately 55%, had one or more occurrences of the RUNX1 matrix in their respective promoter regions. We next investigated the presence of consensus sequences to the RUNX1 “*Position Frequency matrix*” in the promoter of the ECM genes upregulated in DS fetal hearts (Supplementary Table S4) (Conti et al., 2007). *RUNX1* is 1.7 times upregulated in DS hearts when compared with euploid hearts (*p*-value < 0.05). As in the case of mouse genes, we performed this analysis with PSCAN software (Zambelli et al., 2009) and selected the promoter region from −1,000 to +0, with respect to the Transcription Starting Site, of each gene. The output of the computation showed a significant affinity of the RUNX1 matrix to the set of gene promoters under analysis (*p*-value = 3.3e−4) (Supplementary Figure S2). We found that 46 ECM genes, approximately 80% of the genes upregulated in DS fetal hearts, had one or more occurrences of the RUNX1 matrix in their respective promoter regions (Table 3). These results provided us with further insight into the potential role of RUNX1 as a regulator of ECM genes and helped us to choose potential targets that we tested to see if and how they were affected by RUNX1.

**TABLE 3:** ECM genes upregulated in trisomic hearts with consensus sequence for RUNX1 matrix.

Gene	Refseq id	Z-score	Affinity score	Position	Sequence
WNT5B	NM_030775	2.7492	0.9847	−420	GCCTGTGGTTT
ECM2	NM_001197296	2.4800	0.9742	−306	ATCTGTGGTTT
COL6A2	NM_058174	2.0886	0.9589	−642	ACCTGTGGTTT
C1QTNF3	NM_014324	1.9691	0.9543	−994	CTCTGTGGCTT
GPC3	NM_001164617	1.9691	0.9543	−788	CTCTGTGGCTT
AMBP	NM_001633	1.8332	0.9490	−26	GCCTGTGGCTT
APP	NM_001136131	1.8049	09479	−711	GGCTGTGGCTT
FGB	NM_001184741	1.6319	0.9411	−838	TTTTGTGGCTT
FGB	NM_001382759	1.6319	0.9411	−869	TTTTGTGGCTT
PTN	NM_001321386	1.5914	0.9396	−648	TCTTGTGGTTA
TGM4	NM_003241	1.5762	0.9390	−869	GCTTGTGGTCT
ADAMTS5	NM_007038	1.5494	0.9379	−299	CGCTGTGGCTT
GPC4	NM_001448	1.5399	0.9376	−280	CTTTGTGGTTG
ITGB4	NM_001005619	1.4631	0.9346	−734	GGCTGTGGGTT
COL9A2	NM_001852	1.3853	0.9315	−950	GCCTGTGGTCA
CASK	NM_001367721	1.3479	0.9301	−689	GCCTGTGGTTC
CASK	NM_003688	1.3479	0.9301	−169	GCCTGTGGTTC
COL18A1	NM_030582	1.2359	0.9257	−772	CCCTGTGGGTT
LEFTY2	NM_003240	1.1726	0.9232	−839	ACCTGTGGCTT
FGFR2	NM_001144916	1.1293	0.9216	−865	TCCTGTGGTTC
MMP11	NM_005940	0.9403	0.9142	−642	GTCTGTGGTCC
FLRT2	NM_001346146	0.8233	0.9096	−141	GCCTGCGGTTT
ADAMTS3	NM_014243	0.7505	0.9068	−750	CGCTGTGGCCT
APP	NM_001136016	0.7483	0.9067	−862	TTCTGAGGTTT
COL3A1	NM_000090	0.7367	0.9063	−156	TACTGTGGGTT
PTN	NM_002825	0.7114	0.9053	−645	CTCTGAGGTTT
TIMP2	NM_003255	0.7003	0.9048	−593	GGGTGTGGTTA
DCN	NM_001920	0.6967	0.9047	−901	CTCTGTGTTTT
ADAMTS7	NM_014272	0.6925	0.9045	−557	GCCTGTGGGCT
COLEC10	NM_006438	0.6692	0.9036	−823	GCTTGTGGTAT
BGN	NM_001711	0.6562	0.9031	−750	CTCTGTGGGTG
WNT4	NM_030761	0.6385	0.9024	−744	CCTTGTGGCTA
LUM	NM_002345	0.5929	0.9007	−311	ATCTGTGGCTG
HAPLN1	NM_001884	0.5764	0.9000	−347	CTCTGGGGTTT
SPON1	NM_006108	0.5609	0.8994	−813	GCCTGTGTTTT
COL5A1	NM_000093	0.4982	0.8970	−524	GACTGTGGCCT
COL5A1	NM_001278074	0.4982	0.8970	−526	GACTGTGGCCT
TRIL	NM_014817	0.4456	0.8949	−842	TGCTGTGGGCT
COL1A1	NM_000088	0.4409	0.8947	−999	GGCTGTGGCCA
MMP2	NM_001302510	0.4406	0.8947	−655	GCCTGGGGTTT
DCN	NM_133503	0.3596	0.8916	−868	TTTTGTGTTTT
MATN2	NM_001317748	0.3596	0.8916	−228	TTTTGTGTTTT
C1QTNF3	NM_181435	0.3555	0.8914	−130	GCTTGTGGTGT
FBLN1	NM_006487	0.3132	0.8897	−691	ACTTGTGGTTC
FGFR2	NM_001144915	0.3107	0.8897	−39	CTTTGTGGTCC
COL9A3	NM_001853	0.2986	0.8892	−389	GTCTGCGGCTT
APP	NM_000484	0.2274	0.8864	−959	GTTTTTGGTTT
FLRT2	NM_001346143	0.2274	0.8864	−717	GTTTTTGGTTT
VCAN	NM_001126336	0.1975	0.8852	−338	GGCAGTGGTTT
GLG1	NM_001145667	0.1936	0.8851	−203	TGCTGGGGTTT
ACAN	NM_001135	0.1849	0.8848	−973	TGCTGTGGCTC
COL1A2	NM_000089	0.1732	0.8843	−121	AGCTGTGGCTG
COL13A1	NM_001368896	0.1462	0.8832	−441	GCTTGTGGGTG
WNT5B	NM_032642	0.1073	0.8817	−613	GTTTGTGGGTC
COL14A1	NM_021110	0.1040	0.8816	−609	TCGTGTGGTTG
APOA1	NM_001318017	0.0395	0.8791	−562	GACTGAGGTTT
APOA1	NM_001318021	0.0395	0.8791	−234	GACTGAGGTTT
ASPN	NM_017680	0.0088	0.8779	−900	TTTTTTGGTTT

For each submitted gene, it is shown the gene name, the Refseq ID, the z-score, which corresponds to a positive contribution of each input gene to the z-score of the RUNX1 matrix, the oligo affinity score, the position with respect to the TSS of the gene and the oligo sequence. We consider only genes with a positive z-score and an affinity score >0.8.

### *RUNX1* and ECM Gene Expression is Increased in Trisomic Fibroblasts

**FIGURE 1:**
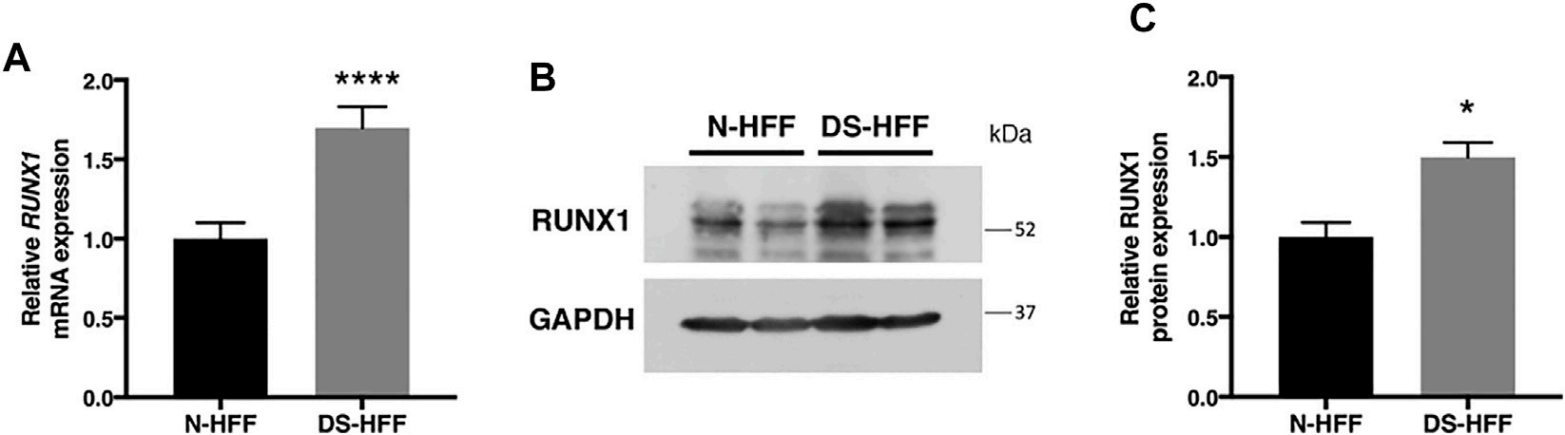
RUNX1 expression is significantly increased in DS-HFFs *vs.* N-HFFs. **(A)** Relative *RUNX1* mRNA expression measured by qRT-PCR and normalized to a reference gene (*GAPDH*). Values represent the average determination from at least 2 qRT-PCR experiments. Results are expressed as relative mean values ± SEM of cell cultures from four euploid and four trisomic samples. **(B)** Representative immunoblot of RUNX1 protein detected in total protein fractions of two N-HFF and two DS-HFF samples. GAPDH was used as a loading control. **(C)** Densitometric analysis from three different experiments. Results are expressed as relative mean values ± SEM from three euploid and three trisomic samples. *p-value < 0.05, ****p-value ≤ 0.0001.

**FIGURE 2:**
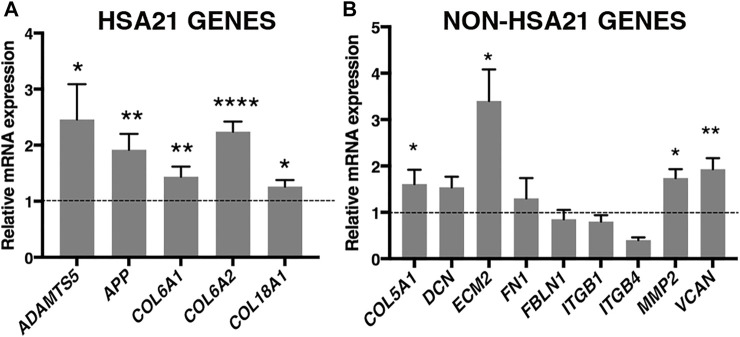
ECM genes are significantly upregulated in trisomic fibroblasts. Relative mRNA expression of Hsa21 **(A)** and non-Hsa21 **(B)** genes coding for ECM proteins, measured by qRT-PCR and normalized to a reference gene (*GAPDH*). Values represent the average determination from at least 2 qRT-PCR experiments. Results are expressed as relative mean values ± SEM of cell cultures from four trisomic samples, compared with a pool of euploid controls (set equal to 1 = dashed line). *p-value ≤ 0.05, **p-value ≤ 0.01, ****p-value ≤ 0.0001.

To validate RUNX1 as a regulator of genes encoding ECM proteins, we used trisomic (DS) and euploid (N) human fetal fibroblasts (HFFs) that have been previously characterized (Piccoli et al., 2013; Izzo et al., 2014; Izzo et al., 2017a; Izzo et al., 2017b; Mollo et al., 2019). We first measured the level of expression of *RUNX1* mRNA by qRT-PCR and found an upregulation of approximately 1.7-fold in trisomic samples compared with a pool of euploid samples (Figure 1A), in agreement with a gene dosage effect. By Western blot analysis we determined that the level of RUNX1 protein was also increased approximately 1.5-fold in trisomic fibroblasts (Figures 1B,C). We next verified, in the same cell culture model, if there was upregulation of ECM genes. To this aim, we chose 14 genes encoding ECM proteins, located or not on Hsa21, which were selected among genes known to have a potential role in cardiac morphogenesis and/or with consensus sequence to the RUNX1 matrix, most of them upregulated in trisomic hearts. We found that the five Hsa21 genes we tested were all significantly upregulated (Figure 2A) while, among the non-Hsa21 genes, four of nine, *COL5A1*, *ECM2*, *MMP2*, and *VCAN*, were significantly upregulated in trisomic fibroblasts (Figure 2B). We therefore concluded that the trisomic cells we analyzed express higher levels of the RUNX1 transcription factor and higher levels of several ECM proteins than their euploid counterparts.

### *RUNX1* Attenuation Decreases the Expression of ECM Genes in Trisomic Fibroblasts

**FIGURE 3:**
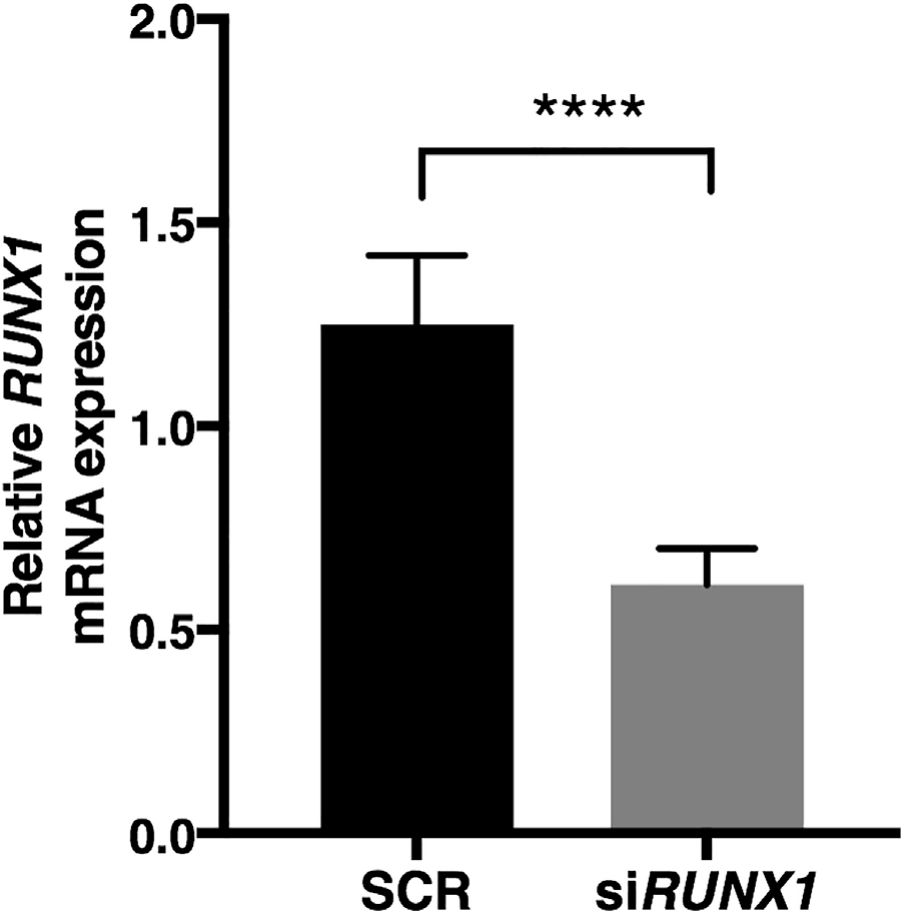
*RUNX1* expression is significantly reduced by specific siRNA. *RUNX1* expression was determined in DS-HFFs transfected with 20 nM si*RUNX1* for 72 h. Values represent the average determination ± SEM from five SCR and the five corresponding *RUNX1*-silenced DS-HFFs carried out in triplicate. ****p-value ≤ 0.0001.

**FIGURE 4:**
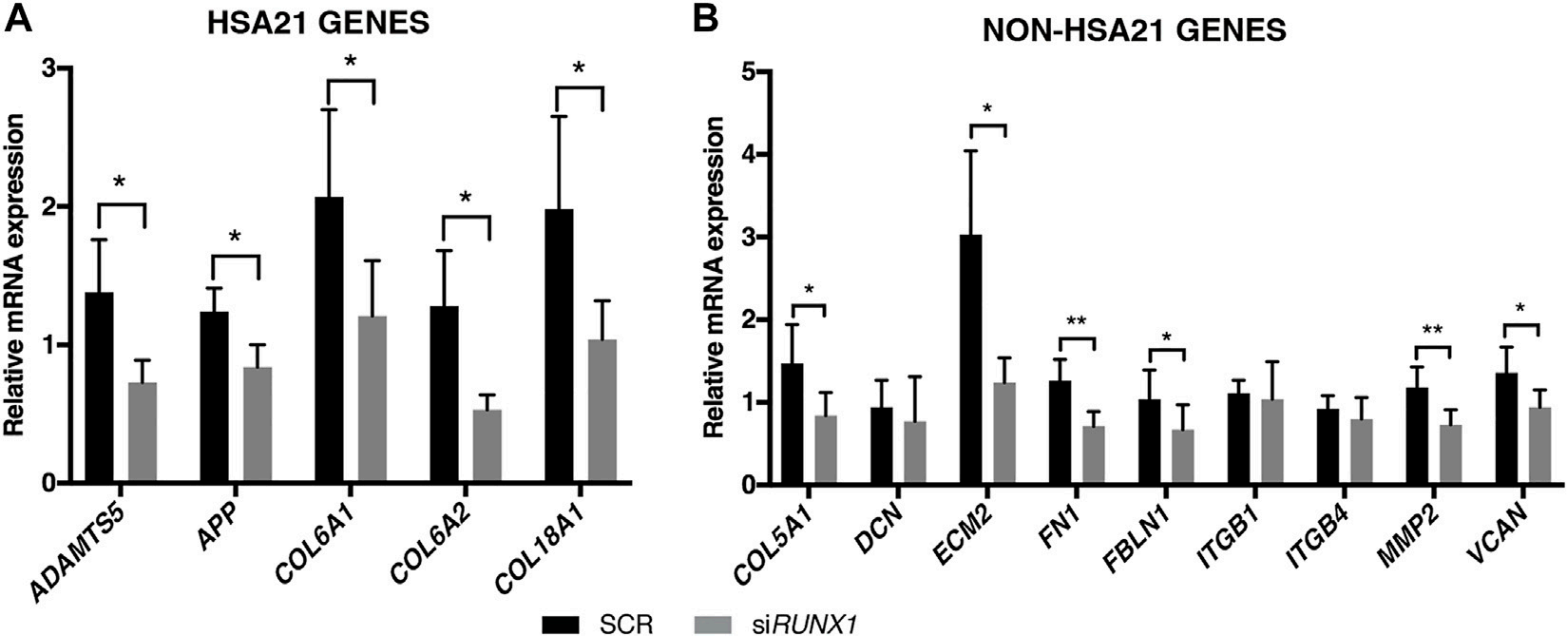
*RUNX1* attenuation decreases ECM gene expression in trisomic fibroblasts. Relative mRNA expression of Hsa21 **(A)** and non-Hsa21 **(B)** genes coding for ECM proteins in DS-HFFs transfected with 20 nM si*RUNX1* for 72 h. Values represent the average determination from at least 2 qRT-PCR experiments. Results are expressed as relative mean values ± SEM of cell cultures from five SCR and the five corresponding *RUNX1*-silenced DS-HFFs carried out in triplicate. *p-value ≤ 0.05, **p-value ≤ 0.01.

To test the hypothesis that overexpression of *RUNX1* affects the expression of ECM genes in trisomic cells, we performed *RUNX1* gene silencing experiments. Based on preliminary evaluations, we selected the timing and doses of a specific SMART pool of siRNAs against *RUNX1* (si*RUNX1*) to be transfected in DS-HFFs. Seventy-two hours after transfection, we determined by qRT-PCR whether *RUNX1* expression was indeed decreased. We also transfected DS-HFFs with non-targeting siRNAs (SCR) as a control. We observed a significant reduction (at least 2-fold) in *RUNX1* expression in DS-HFFs after transfection with the specific siRNA pool (Figure 3). We then evaluated the expression of those ECM genes that we hypothesized might be regulated by RUNX1. We first examined the effect of silencing on the Hsa21 genes and found that the expression of each gene was significantly decreased (Figure 4A). In contrast, among the nine non-Hsa21 genes previously considered, there was a significant reduction in the mRNA levels of six ones (*COL5A1*, *ECM2*, *FN1*, *FBLN1*, *MMP2*, and *VCAN*) (Figure 4B). It is of note that, although *FN1* and *FBLN1* did not show significant changes in expression between N-HFFs and DS-HFFs, they were still modulated by *RUNX1* silencing.

### Collagen IV is Present at Higher Levels in Trisomic Cells and Culture Media

**FIGURE 5:**
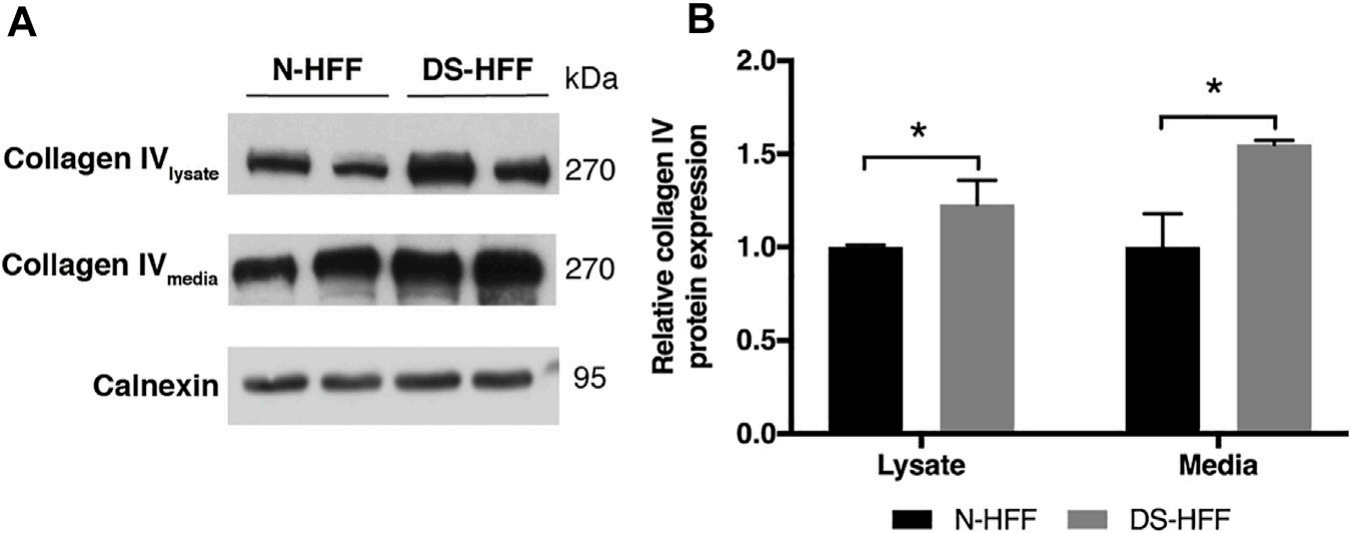
Trisomic samples show a significant increase of collagen IV protein in cell lysates and culture media. **(A)** Representative immunoblotting of collagen IV protein detected in cell lysates and media of three N-HFF and three DS-HFF samples. Calnexin was used as a loading control. **(B)** Densitometric analysis from three different experiments. Results are expressed as relative mean values ± SEM from three N-HFF and three DS-HFF samples. *p-value ≤ 0.05.

**FIGURE 6:**
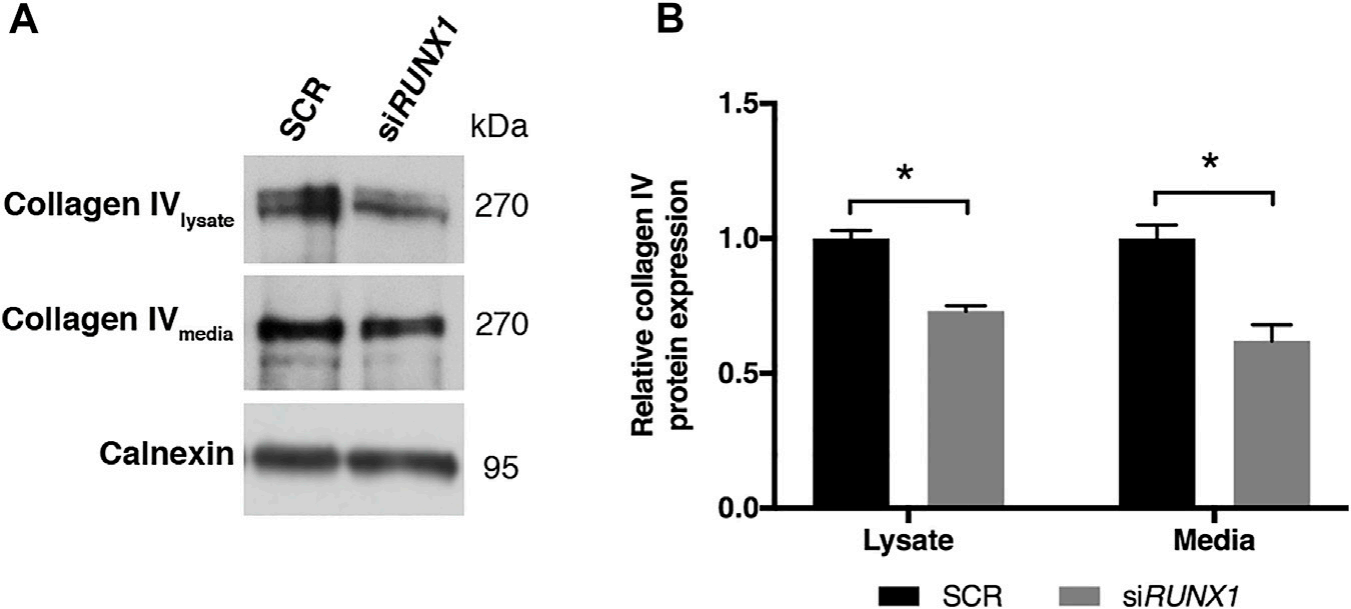
*RUNX1* silencing leads to a significant decrease of collagen IV protein in media of silenced trisomic samples. **(A)** Representative immunoblotting of collagen IV protein detected in cell lysates and culture media of SCR and si*RUNX1*-treated DS-HFF samples. Calnexin was used as a loading control. **(B)** Densitometric analysis from two different experiments. In each experiment we used two out of the three DS-HFFs samples of Figure 5. Results are expressed as relative mean values ± SEM from two SCR and the two corresponding *RUNX1*-silenced DS-HFFs. *p-value ≤ 0.05.

Collagen IV is a major constituent of basement membrane and is highly expressed in skin fibroblasts. It consists of two alpha1(IV) chains (coded by *COL4A1*) that combine with an alpha2(IV) chain (coded by *COL4A2*) to make the complete collagen IV molecule. The *COL4A1* promoter region contains several putative binding sites for RUNX1 and is transcriptionally regulated by the same transcription factor in different cell lines (Wang et al., 2020). *COL4A1* and *COL4A2* genes are consistently upregulated in DS cells and tissues (Vilardell et al., 2011) but not in fetal hearts (Conti et al., 2007). We found that *COL4A1* mRNA expression decreased after *RUNX1* silencing (Supplementary Figure S3). We tested if collagen IV protein is regulated by RUNX1 in trisomic fibroblasts. We evaluated by Western blot analysis the level of this protein in cell lysates and culture media of DS-HFF and N-HFF. We detected a significantly higher amount of collagen IV protein in cell lysates and culture media from trisomic cultures (Figure 5). We then aimed at determining whether *RUNX1* silencing affects collagen IV expression and its rate of secretion in trisomic cells. We detected by immunoblotting a significant reduction of collagen IV in cell lysates and culture media of si*RUNX1*-transfected trisomic fibroblasts (Figure 6). These results suggest that *RUNX1* overexpression may contribute to the overexpression of collagen IV in trisomic cells.

### Trisomic Fibroblasts Migrate More Slowly Than Euploid Ones

**FIGURE 7:**
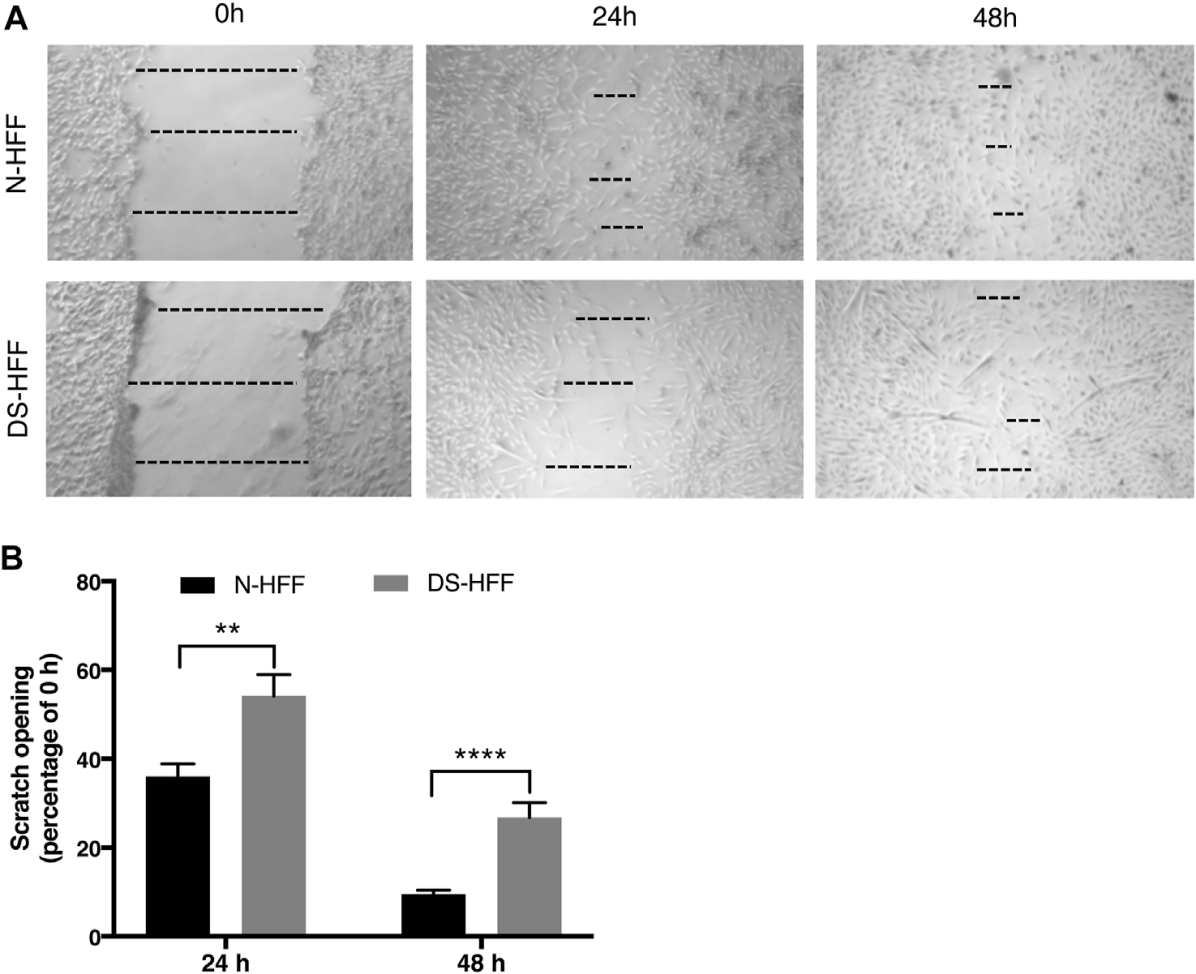
Cell migration is compromised in DS-HFFs. The rate of migration was measured by quantifying, in different points, the distance of the area lacking cells. **(A)** Representative images of N-HFF and DS-HFF scratch opening at 0, 24, and 48 h. The dotted lines define the area lacking cells (wound area, ImageJ). **(B)** Scratch opening quantification. Mean values ± SEM from four euploid and four trisomic samples are represented as percentage of scratch opening at 0 h. **p-value ≤ 0.01, ****p-value ≤ 0.0001.

Upregulation of ECM genes can affect cell-substrate interactions and thus alter cell migration properties. We performed a wound healing assay by scratching the cell monolayer to compare the migratory capabilities of trisomic cells with those of non-trisomic cells. Images acquired at 24 h showed a significant difference in the opening of the scratch between N-HFFs and DS-HFFs. The distance between the edges was greater in DS-HFFs (Figure 7), indicating a lower migratory potential of trisomic fibroblasts. An even more significant difference was observed when we analyzed the images acquired at 48 h (Figure 7). Based on these results, we concluded that trisomic fibroblasts migrate more slowly than euploid ones, possibly due to the different composition of the ECM that might affect their adhesiveness.

### *RUNX1* Attenuation Increases Cellular Migration in Trisomic Fibroblasts

**FIGURE 8:**
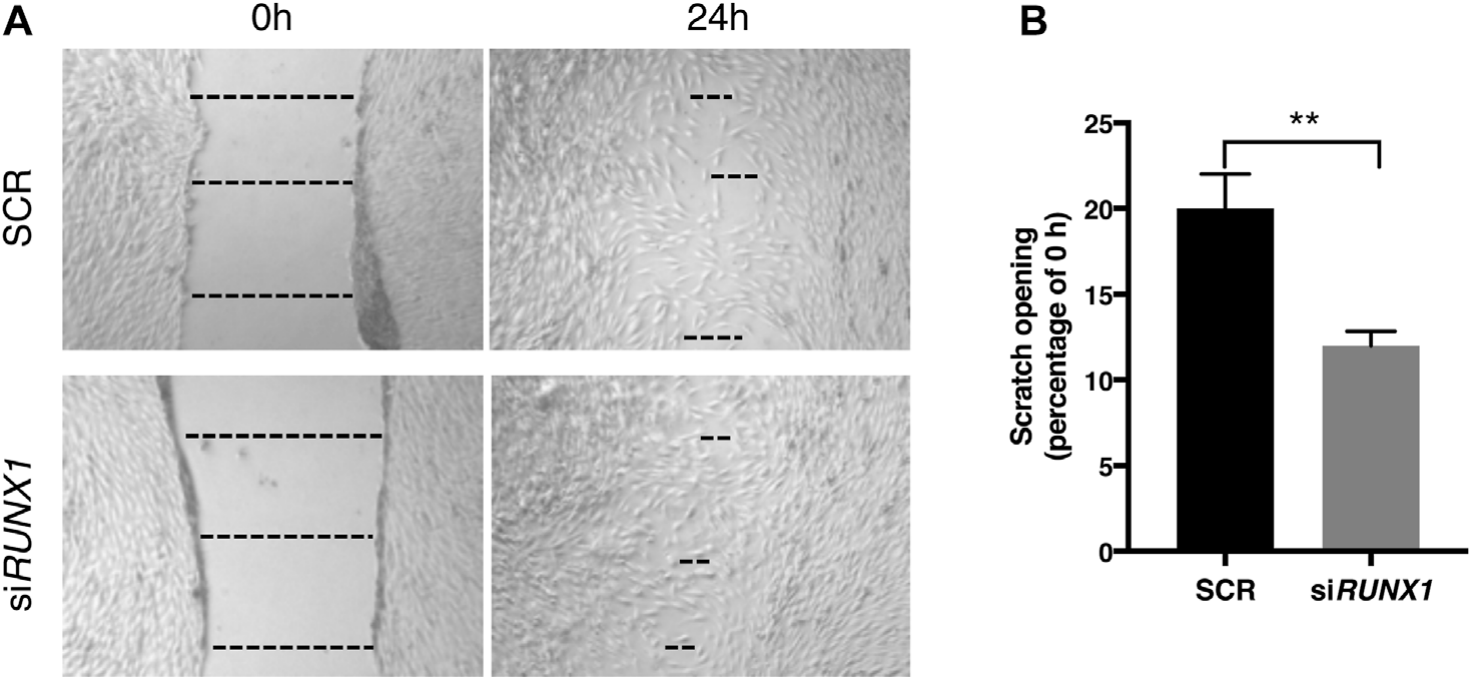
*RUNX1* silencing influences trisomic cell migration properties. The rate of migration was measured by quantifying, in different points, the distance of the area lacking cells. **(A)** Representative images of scratch opening of SCR and si*RUNX1*-treated DS-HFF at 0 and 24 h. The dotted lines define the area lacking cells (wound area, ImageJ). **(B)** Scratch opening quantification. Mean values ± SEM from four SCR- and four si*RUNX1*-treated trisomic samples are represented as percentage of scratch opening at 0 h. **p-value ≤ 0.01.

*RUNX1* overexpression in trisomic cells likely causes an increase in the expression of ECM genes, which in turn might affect cell migration. We tested whether attenuation of *RUNX1* by siRNA, together with the resulting decrease in ECM gene expression, could affect the migration properties of trisomic cells. We performed the wound healing assay in DS-HFFs treated with 20 nM si*RUNX1* for 72 h. We found that at 24 h there was already a significant difference in the scratch opening between silenced and control cultures. In particular, trisomic cells treated with 20 nM si*RUNX1* migrated faster than the control cells, with a 12% opening compared to 20% for controls (Figure 8). These results suggest that *RUNX1* silencing can affect the migration capacity of trisomic cells, possibly by acting on ECM composition and/or remodeling.

## Discussion

Dysregulated expression and/or organization of ECM components in DS is responsible for altered morphogenesis and causes organ malformations, including CHD (Gittenberger-De Groot et al., 2003; Garcia et al., 2010; Danopoulos et al., 2021). In this study we propose that the upregulation of *RUNX1*, a transcription factor that maps to Hsa21, contributes to the overexpression of ECM genes in trisomic cells and that this overexpression is responsible for a decreased migration of trisomic fibroblasts.

### RUNX1 is a Regulator of ECM Gene Expression

The overexpression of ECM genes in DS has been hypothesized since 1998 when ultrastructural findings showed that an extracellular precipitate containing glycosaminoglycans is regularly present in the skin of trisomy 21 human fetuses (Von Kaisenberg et al., 1998). Global overexpression of ECM genes was demonstrated by transcriptome profiling of heart tissues from fetuses with trisomy 21 (Conti et al., 2007). These findings are also confirmed by a meta-analysis of DS expression data sets (Vilardell et al., 2011) where the 324 upregulated genes were significantly enriched for ECM Cell Component GO category.

Here we attempted to find which Hsa21 gene(s) might affect the expression of ECM genes in DS tissues. Several lines of evidence support the role of RUNX1 as a regulator of ECM genes: 1) *Runx1* is the only gene causing the upregulation of ECM genes from overexpression experiments of 32 Hsa21 transcription factors and regulators in mouse embryo cells (De Cegli et al., 2010); 2) GSEA analysis of four experiments (Table 2) in which *RUNX1* gene expression was modulated, demonstrated that the ECM Cell Component GO category is always affected by *RUNX1* modulation in different organisms and conditions, despite few genes being consistently dysregulated across all four sets; 3) there is a highly significant affinity of the RUNX1 Position Frequency matrix to the ECM genes upregulated in DS fetal hearts. About 80% of them showed one or more occurrences of the RUNX1 matrix in the respective promoter regions; 4) the 324 genes consistently upregulated from 45 DS data sets are enriched in RUNX1 motifs as revealed by promoter analysis (Vilardell et al., 2011).

It must be noted, however, that the overlap among the different lists of genes belonging to the ECM category extracted from the datasets we analyzed is not very high. We think that this result is expected considering the differences in the cell type analyzed, the species and the treatments (Michaud et al., 2008; Wotton et al., 2008; Lie-A-Ling et al., 2014; Barutcu et al., 2016). However, each approach independently provides a large source of data to identify RUNX1 targets according to *RUNX1* gene dosage, and their combination is powerful because it has allowed the prioritization of novel candidate genes.

### RUNX1 Contributes to the Overexpression of ECM Genes in Trisomic Fibroblasts

To validate the hypothesis that RUNX1 plays a role in modulating the expression of ECM genes, we measured the mRNA levels of some of these genes in cells with Hsa21 trisomy before and after attenuation of *RUNX1* expression. Primary cultures of fibroblasts were chosen as a model because these cells constantly perform ECM remodeling through synthesis, degradation, reassembly, and chemical modification of ECM proteins (Bonnans et al., 2014). Both mRNA and protein expression of RUNX1 were upregulated in trisomic fibroblasts, as expected from the gene dosage effect. *RUNX1* was among the 30 genes with the highest dosage effects in the meta-analysis of 45 different DS studies on humans and mice at the transcriptome and proteome level (Vilardell et al., 2011). Its upregulation was demonstrated also in DS embryonic stem cells, embryoid bodies and neural progenitor cells (Halevy et al., 2016).

Fourteen ECM genes were tested to assess if they were RUNX1 targets. They were chosen according to their behavior after RUNX1 modulation and/or because of their suggested role in cardiogenesis. Five of these genes map to Hsa21 and were found to be significantly upregulated in trisomic cells possibly because of gene dosage. However, since they have predicted binding sites for RUNX1, it was intriguing to check their possible dependence upon this transcription factor. After *RUNX1* attenuation by siRNA, all the Hsa21 genes showed a significant decrease in their expression proving to be at least partially affected by *RUNX1* expression levels. Interestingly, *APP* is significantly reduced after *RUNX1* silencing. This result could be further explored as it has been demonstrated a role of *RUNX1* in neurogenesis and neurite outgrowth (Halevy et al., 2016; Fukui et al., 2018) and a detrimental role for APP protein has been suggested from early stages of DS neurogenesis (Sobol et al., 2019). The modulation of *COL6A1* and *COL6A2* expression after *RUNX1* silencing could explain the causal link between trisomy 21 and an increased incidence of Hirschsprung’s disease in DS (Heuckeroth, 2015). It has been demonstrated that an excess of collagen VI has an inhibitory effect on the migration of enteric neural crest-derived cells, responsible for the formation of enteric nervous system (Soret et al., 2015). Six out of the nine ECM genes mapping to other chromosomes (*COL5A1*, *ECM2*, *FN1*, *FBLN1*, *MMP2*, and *VCAN*) were also affected by *RUNX1* attenuation. Two of these genes, *FN1* and *FBLN1*, which did not exhibit any significant differential expression between trisomic and non-trisomic cells, were similarly downregulated upon *RUNX1* attenuation. On the other hand, the integrin *ITGB4*, a target of RUNX1 in myeloid cells (Phillips et al., 2018), was poorly expressed in trisomic fibroblasts and did not appear to be affected at all by *RUNX1* modulation in this model. We do not have an explanation for the behavior of these genes. However, it should be considered that an intricate network controls the expression of all these genes and that *RUNX1* is only one among the many players in the game. Moreover, the overexpression of ECM genes might be a direct, as well as an indirect, consequence of higher activity of the RUNX1 transcription factor.

We demonstrated that collagen IV protein levels were increased in trisomic fibroblasts and media, and decreased upon *RUNX1* attenuation. *COL4A1* expression is regulated by RUNX1 in different cell types. Overexpression of this transcription factor in hepatocellular carcinoma cells dramatically elevates COL4A1 expression while its knockdown in SMMC7721 and SK-Hep1 cells significantly decreases the COL4A1 expression level (Wang et al., 2020). COL4A1 is only 127 bp apart from COL4A2 on chromosome 13q34 in a head-to-head arrangement and they share a bidirectional promoter. The same transcription factors can affect the expression of both genes. As a matter of fact, *COL4A2* expression is positively correlated with that of *COL4A1* (Pollner et al., 1997; Kuo et al., 2012).

### RUNX1 Expression Modulates Cell Migration

Cell migration experiments in this study showed that trisomic fibroblasts migrate more slowly than controls. Decreased cellular migration and increased cellular adhesiveness of fibroblasts from the endocardial cushions of the atrioventricular (AV) canal in DS may result in AV canal defects (Kurnit et al., 1985). Defective migration was demonstrated in a murine DS cell model in which a supernumerary Hsa21 was expressed (Delom et al., 2009; Nižetić and Groet, 2012). *RUNX1* attenuation by siRNA transfection in human trisomic fibroblasts causes an increment of cell migration dynamics. This could be due to a change in the expression of ECM genes, which may code for the substrates on which the cells move, or of other related genes, such as integrins, which mediate the interaction with the ECM (Lauffenburger and Horwitz, 1996; Conway and Jacquemet, 2019).

Our data indicate that, among the ECM genes that are affected by RUNX1 in cultured fibroblasts, there are those for members of the collagen family, like COL5A1, COL6A1, COL6A2, and COL18A1; proteins that contribute to ECM assembly, like ECM2; proteoglycans, like VCAN; and disintegrins and metalloproteinases, like ADAMTS5 and MMP2. RUNX1 regulates the expression of several members of the integrin family, such as ITGA6 and ITGB4 in myeloid cells (Phillips et al., 2018). It may indirectly regulate cell–matrix or cell–cell adhesion contacts as well as signaling and transcriptional programs of the cell via the α6β4 receptor (Phillips et al., 2018), which is also known to regulate collective migration of epithelial cells (Colburn and Jones, 2017). Altered expression of integrins has been demonstrated in cells or leukemic samples in which *RUNX1* is disrupted (Valk et al., 2004; Tanaka et al., 2012), suggesting that many members of this family may be regulated by RUNX1. In another approach, the integration of a special ChIP-seq with matching transcriptome data (reported in our study as SET4), revealed that RUNX1 binds to and upregulates the expression of genes involved in cell adhesion and migration, including components of the integrin signaling pathway as well as downstream targets (Lie-A-Ling et al., 2014). Finally, ablation of *RUNX1* in DS-NPCs revealed that nearly 70% of downregulated genes were putative targets of RUNX1 and were involved in neuron/cell migration and regulation of cell growth (Halevy et al., 2016).

### Role of RUNX1 and ECM Proteins in Cardiac Morphogenesis

The ECM plays a critical role in the development of the heart, in which the cardiac jelly, an acellular and ECM-rich space that separates the myocardial and endocardial cell layers in the primitive heart (Wirrig et al., 2007), has a pivotal role in heart septation and valvulogenesis. Signaling events originating in the myocardium cause cells in the endocardium to undergo an epithelial to mesenchymal transformation (EMT) and migrate into cardiac jelly (Shelton and Yutzey, 2007) forming the endocardial cushions. The endocardial cushions will then elongate and undergo ECM remodeling in order to form septa and functionally mature valves with increased organization and complexity of the ECM (Shelton and Yutzey, 2007). It is noteworthy that the heart frequently undergoes morphogenesis defects in DS and that the large majority of these are endocardial cushion defects. In fact, about 50% of DS live births have CHD (Urbano, 2010; Stoll et al., 2015) and, of these, more than 80% are endocardial cushion defects (Maslen et al., 2006). Thus, it seems plausible that alterations in the expression of ECM genes might play a role in DS-CHD.

It is also notable that the *Runx1* gene is included in the 3.7 Mb minimal critical region for DS-CHD in mice (Liu et al., 2014). Only two others transcription factors, *Sim2* and *Ripply3/Dscr6*, map to this region but their overexpression does not seem to affect the expression of ECM genes (De Cegli et al., 2010). A quantitative analysis of copy number variations (CNVs) in subjects with cardiac malformations revealed that *RUNX1* was among the most significantly enriched genes involved in genomic duplications associated with CHD. In particular, gains involving *RUNX1* were more frequent in subjects with AV septal defects and tetralogy of Fallot, which are endocardial cushion defects frequently observed in DS (Tomita-Mitchell et al., 2012).

Several ECM proteins, regulated by RUNX1 and upregulated in DS fetal hearts, could play a role in determining cardiac defects. Among these there is VCAN, which binds to several ECM components influencing cell adhesion, proliferation, migration, and survival. Its role is necessary for cardiac cushion formation, AV valve development, ventricular septation, and outflow tract development (Chan et al., 2010). VCAN is cleaved by MMPs and members of the ADAMTS family (Kern et al., 2006). This process is necessary for the development of the AV cushions, outflow tract, and trabeculae (Kern et al., 2006; Kern et al., 2007; Stankunas et al., 2008). The dysregulation of proteins from the ADAMTS family is supposed to compromise this process. VCAN, as well as a variety of other ECM molecules, binds to FBLN1 (Lee et al., 2005; Kern et al., 2006; Cooley et al., 2008), a protein that is expressed throughout the ECM of the AV cushions (Kern et al., 2006). Other ECM components that affect cardiogenesis are collagens, like collagen VI, whose expression has been documented in the developing AV cushions and the adult AV valves of several species (Bonaldo et al., 1998; Klewer et al., 1998; Gittenberger-De Groot et al., 2003; Kruithof et al., 2007), and *Col5a1*, whose knockout decreases collagen fibril formation in mice and is embryonic lethal because of cardiovascular insufficiency (Wenstrup et al., 2004). *COL5A1* was affected by *RUNX1* modulation in our experiments and consistently in several datasets we have analyzed (Michaud et al., 2008; Lie-A-Ling et al., 2014; Barutcu et al., 2016). Finally, FN1, a multi-domain ECM protein that interacts with multiple integrins, proteoglycans, collagens, and fibrins (Pankov and Yamada, 2002), is expressed early in embryonic development (FFrench-Constant and Hynes, 1989; George et al., 1993). As cardiac development progresses, it is expressed in the dorsal aortae, pharyngeal arch arteries, and endocardium (FFrench-Constant and Hynes, 1989; Astrof et al., 2007), and in the mesenchyme of the endocardial cushions, where it is required for EMT-mediated development (Mjaatvedt et al., 1987; Icardo et al., 1992).

It seems conceivable that even a small dysregulation of multiple ECM proteins involved in cardiogenesis may have profound effects on the proper formation of the AV septum and outflow tract, resulting in cardiac defects such as those observed in DS. Although the transcriptome profile of the heart during development, in fetuses with chromosome 21 trisomy, has been shown to be characterized by a significant increase in expression of RUNX1 and many ECM genes (Conti et al., 2007), the demonstration that in hearts of fetuses that develop CHD the increase in expression is higher than in those that do not develop CHD is still missing. It is likely that variations in RUNX1 and ECM levels should be sought in those precise areas of the heart that undergo specific alterations in DS. Currently, there is no evidence to suggest that RUNX1 is the only factor involved in the development of CHD in DS.

## Conclusion

These results suggest that the overexpression of *RUNX1* in trisomic tissues, possibly due to a gene dosage effect, causes the upregulation of ECM genes, which in turn may affect cellular functions and organ morphogenesis during development. The heart is an organ that frequently undergoes morphogenetic alterations in DS. CHD in DS is mostly represented by endocardial cushion defects and endocardial cushions mainly consist of ECM proteins. We demonstrate that *RUNX1*, which maps to the minimal critical region responsible for CHD in mice, is a main controller of ECM gene expression. Even a small alteration in the expression level of many ECM genes can likely perturb the cardiac morphogenesis process. Attenuation of the expression of *RUNX1* can impair the upregulation of ECM genes and rescue cell migration defects. The demonstration that during heart formation in trisomic individuals who develop CHD, compared with those who do not, there is overexpression of *RUNX1* and ECM genes, possibly in specific areas of the heart, would be a useful finding to support a substantial role of RUNX1 in the development of CHD in DS.

## Data Availability

Publicly available datasets were analyzed in this study. This data can be found here: https://www.ncbi.nlm.nih.gov/geo/query/acc.cgi?acc=GSE19836 Gene Expression Omnibus (GEO) GSE19836. All other data in the study are available on request from the corresponding author.
