# Supplementary material for: Overexpression of the Hsa21 Transcription Factor RUNX1 Modulates the Extracellular Matrix in Trisomy 21 Cells
Source: Front Genet. 2022 Mar 10;13:824922. doi: 10.3389/fgene.2022.824922 (PMC8960062; doi:10.3389/fgene.2022.824922)
Supplement: Supplementary file 1 [file DataSheet2.docx]

**Supplementary Figure 1. Detailed information of the RUNX1 matrix displayed by PSCAN analysis on mouse GO ECM genes.** The top of the panel shows the info matrix (left) and the Position Frequency matrix (right) which represents the frequency with which each nucleotide appears at each position in the alignment. The sample mean score is the average score in the input set compared to the mean and standard deviation of the background (green area). Below this information, the interface shows statistics for the array on the input set, along with an input form for comparing results on different input sets. At the bottom, the Runx1 matrix logo is displayed.


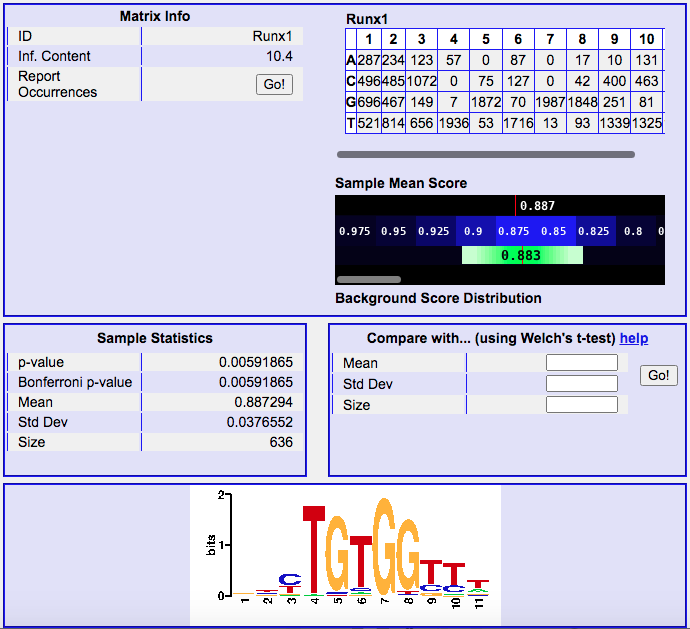


**Supplementary Figure 2. Detailed information of the RUNX1 matrix displayed by PSCAN analysis on ECM genes upregulated in fetal hearts.** The top of the panel shows the info matrix (left) and the Position Frequency matrix (right). which represents the frequency with which each nucleotide appears at each position in the alignment. The sample mean score is the average score in the input set compared to the mean and standard deviation of the background (green area). Below this information. the interface shows statistics for the array on the input set. along with an input form for comparing results on different input sets. At the bottom the Runx1 matrix logo is displayed.


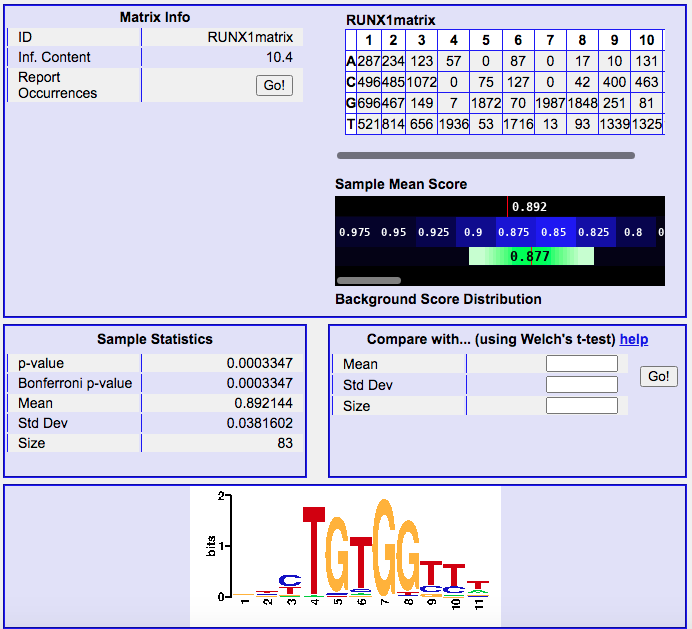


**Supplementary Figure 3.** ***RUNX1* attenuation decreases *COL4A1* gene expression in trisomic fibroblasts.**


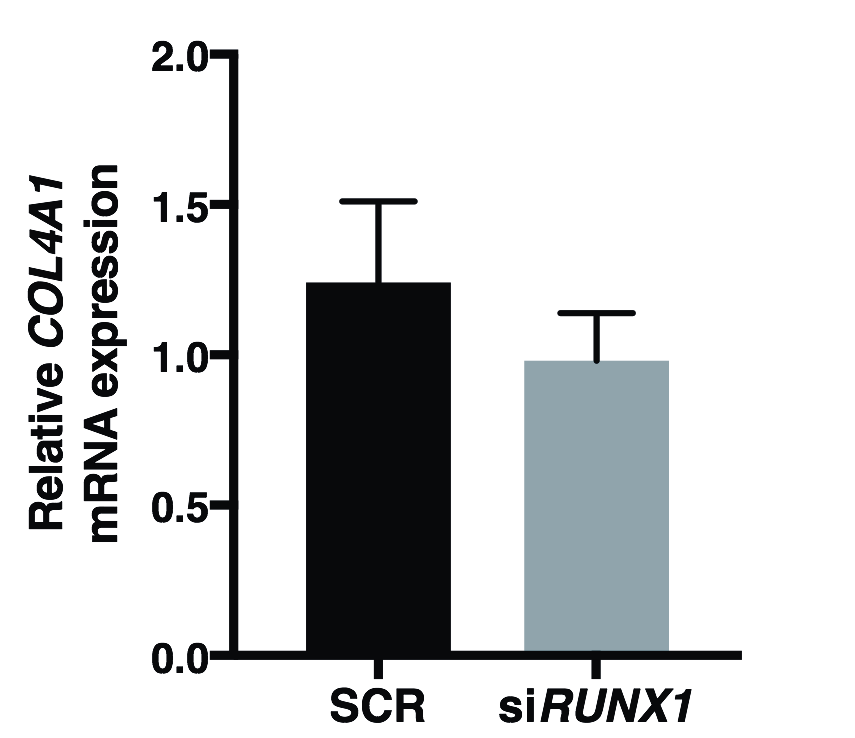


Relative mRNA expression of *COL4A1* in DS-HFFs transfected with 20nM si*RUNX1* for 72 h. Values represent the average determination from 2 qRT-PCR experiments. Results are expressed as relative mean values ± SEM of cell cultures from two SCR and the two corresponding *RUNX1*-silenced DS-HFFs carried out in triplicate.
