# Supplementary material for: Overexpression of the Hsa21 Transcription Factor RUNX1 Modulates the Extracellular Matrix in Trisomy 21 Cells
Source: Front Genet. 2022 Mar 10;13:824922. doi: 10.3389/fgene.2022.824922 (PMC8960062; doi:10.3389/fgene.2022.824922)
Supplement: Supplementary file 2 [file DataSheet1.docx]

Supplementary Material

# Supplementary Table 1. Genes belonging to extracellular matrix category upregulated after *Runx1* overexpression in GSE19836 dataset (De Cegli et al., 2010). We selected genes with logFC > 0.3 and adjusted P-value < 0.01.

| **Gene Symbol** | **logFC** | | **Gene Title** | |
| --- | --- | --- | --- | --- |
| *Adamts4* | 1.8189 | | a disintegrin-like and metallopeptidase (reprolysin type) with thrombospondin type 1 motif. 4 | |
| *Anxa1* | 1.8068 | | annexin A1 | |
| *Anxa2* | 1.2834 | | annexin A2 | |
| *Anxa2* | 0.7152 | | annexin A2 | |
| *Anxa4* | 0.3870 | | annexin A4 | |
| *Anxa5* | 0.5559 | | annexin A5 | |
| *Bmp7* | 1.0405 | | bone morphogenetic protein 7 | |
| *Cd151* | 0.5910 | | CD151 antigen | |
| *Cd151* | 0.6122 | | CD151 antigen | |
| *Chad* | 2.3546 | | chondroadherin | |
| *Coch* | 0.9890 | | cochlin | |
| *Col5a1* | 0.7582 | | collagen. type V. alpha 1 | |
| *Col7a1* | 0.5012 | | collagen. type VII. alpha 1 | |
| *Comp* | 1.0659 | | cartilage oligomeric matrix protein | |
| *Ctsb* | 0.6427 | | cathepsin B | |
| *Ctsl* | 1.2581 | | cathepsin L | |
| *Eln* | 1.5402 | | elastin | |
| *Fn1* | 0.8291 | | fibronectin 1 | |
| *Gpc1* | 1.1272 | | glypican 1 | |
| *Gsto1* | 1.8754 | | glutathione S-transferase omega 1 | |
| *Ihh* | 0.9705 | | Indian hedgehog | |
| *Lgals1* | 1.1155 | | lectin. galactose binding. soluble 1 | |
| *Ltbp1* | 1.2476 | | latent transforming growth factor beta binding protein 1 | |
| *Ltbp3* | 0.8160 | | latent transforming growth factor beta binding protein 3 | |
| *Matn1* | 1.6085 | | matrilin 1. cartilage matrix protein | |
| *Mmp17* | 0.8044 | | matrix metallopeptidase 17 | |
| *Mmp9* | 2.5223 | | matrix metallopeptidase 9 | |
| *Mmp9* | 1.9822 | | matrix metallopeptidase 9 | |
| *Npnt* | 2.9185 | | nephronectin | |
| *Npnt* | 2.5249 | | nephronectin | |
| *Serpinb9* | 0.8168 | | serine (or cysteine) peptidase inhibitor. clade B. member 9 | |
| *Sfrp1* | 0.7045 | | secreted frizzled-related protein 1 | |
| *Sfrp1* | 1.0145 | | secreted frizzled-related protein 1 | |
| *Smoc2* | 0.9291 | | SPARC related modular calcium binding 2 | |
| *Smoc2* | 0.6954 | | SPARC related modular calcium binding 2 | |
| *Sparc* | 0.9540 | | secreted acidic cysteine rich glycoprotein | |
| *Tgm2* | 1.4227 | | transglutaminase 2. C polypeptide | |
| *Tgm2* | 1.3168 | | transglutaminase 2. C polypeptide | |
| *Thbs2* | 1.0680 | thrombospondin 2 | |  |
| *Timp1* | 0.8924 | tissue inhibitor of metalloproteinase 1 | |  |
| *Timp3* | 1.1709 | tissue inhibitor of metalloproteinase 3 | |  |
| *Timp3* | 1.5932 | tissue inhibitor of metalloproteinase 3 | |  |
| *Tinagl1* | 1.1464 | tubulointerstitial nephritis antigen-like 1 | |  |
| *Vasn* | 1.7605 | vasorin | |  |
| *Vit* | 0.6819 | vitrin | |  |

| **Enrichment results of SET1 downregulated genes for GO Cellular component categories** | | | | |
| --- | --- | --- | --- | --- |
| **Gene Set** | **Description** | **Observed genes** | **Enrichment Ratio** | **Adjusted P-value** |
| GO:0015629 | actin cytoskeleton | 80/439 | 1.68 | 1.2e-6 |
| GO:0031252 | cell leading edge | 73/403 | 1.67 | 4.7e-6 |
| GO:0070161 | anchoring junction | 67/324 | 1.91 | 8.5e-4 |
| GO:0016323 | basolateral plasma membrane | 45/240 | 1.73 | 1.4e-4 |
| **GO:0031012** | **extracellular matrix** | **72/441** | **1.51** | **1.8e-4** |
| GO:1990204 | oxidoreductase complex | 24/103 | 2.16 | 1.9e-4 |
| GO:0016234 | inclusion body | 19/75 | 2.34 | 2.8e-4 |
| GO:0009897 | external side of plasma membrane | 61/371 | 1.52 | 4.7e-4 |
| GO:0098589 | membrane region | 76/389 | 1.81 | 1.3e-3 |
| GO:0030055 | cell-substrate junction | 43/182 | 2.19 | 4.5e-3 |

**Supplementary Table 2.** **Cellular component GO categories enriched in dysregulated genes after RUNX1 modulation.** Dysregulated genes from SET1 to SET4 were analyzed by Webgestalt software for GSEA in Cellular component categories. The tables indicate for each GO category: the number of observed genes out of all genes belonging to such category represented on the platform used for the analysis; the enrichment ratio of observed to expected genes; the adjusted P-value.

| **Enrichment results of SET2 downregulated genes for GO Cellular component categories** | | | | |
| --- | --- | --- | --- | --- |
| **Gene Set** | **Description** | **Observed genes** | **Enrichment**  **Ratio** | **Adjusted**  **P-value** |
| **GO:0031012** | **extracellular matrix** | **28/496** | **2.77** | **8.2e-7** |
| GO:0005581 | collagen trimer | 8/87 | 4.52 | 3.7e-4 |
| GO:0042383 | sarcolemma | 9/134 | 3.30 | 1.6e-3 |
| GO:0043235 | receptor complex | 17/396 | 2.11 | 2.9e-3 |
| GO:0098636 | protein complex involved in cell adhesion | 4/35 | 5.62 | 5.3e-3 |
| GO:0005788 | endoplasmic reticulum lumen | 13/306 | 2.09 | 9.6e-3 |
| GO:0008180 | COP9 signalosome | 3/34 | 4.34 | 3.1e-2 |
| GO:0097060 | synaptic membrane | 14/430 | 1.60 | 5.5e-2 |
| GO:0005911 | cell-cell junction | 14/441 | 1.56 | 6.5e-2 |

| **Enrichment results of SET3 upregulated genes for GO Cellular component categories** | | | | |
| --- | --- | --- | --- | --- |
| **Gene Set** | **Description** | **Observed genes** | **Enrichment**  **Ratio** | **Adjusted**  **P-value** |
| **GO:0031012** | **extracellular matrix** | **10/424** | **3.73** | **2.8e-4** |
| GO:0098636 | protein complex involved in cell adhesion | 3/31 | 15.30 | 9.5e-4 |
| GO:0031225 | anchored component of membrane | 5/179 | 4.42 | 5.3e-3 |
| GO:0043235 | receptor complex | 7/359 | 3.08 | 7.1e-3 |
| GO:0005770 | late endosome | 4/214 | 2.95 | 4.6e-2 |
| GO:0009897 | external side of plasma membrane | 5/341 | 2.32 | 6.3e-2 |
| GO:0005581 | collagen trimer | 2/73 | 4.33 | 7.7e-2 |
| GO:0016234 | inclusion body | 2/73 | 4.33 | 7.7e-2 |
| GO:0016605 | PML body | 2/93 | 3.40 | 1.1e-1 |
| GO:0005796 | Golgi lumen | 1/23 | 6.87 | 1.3e-1 |

| **Enrichment results of SET4 downregulated genes for GO Cellular component categories** | | | | |
| --- | --- | --- | --- | --- |
| **Gene Set** | **Description** | **Observed genes** | **Enrichment**  **Ratio** | **Adjusted**  **P-value** |
| **GO:0031012** | **extracellular matrix** | **21/441** | **3.83** | **9.4e-4** |
| GO:0043235 | receptor complex | 14/371 | 3.03 | 1.9e-4 |
| GO:0016323 | basolateral plasma membrane | 14/240 | 3.01 | 2.9e-3 |
| GO:0015629 | actin cytoskeleton | 13/439 | 2.38 | 3.1e-3 |
| GO:0000792 | heterochromatin | 5/88 | 4.57 | 4.7e-3 |
| GO:0098636 | protein complex involved in cell adhesion | 3/31 | 7.78 | 6.5e-3 |
| GO:0060076 | excitatory synapse | 4/71 | 4.53 | 1.1e-2 |
| GO:0097060 | synaptic membrane | 12/484 | 1.99 | 1.7e-2 |
| GO:0030055 | cell-substrate junction | 6/182 | 2.65 | 2.5e-2 |
| GO:0000790 | nuclear chromatin | 9/349 | 2.07 | 2.9e-2 |

**Supplementary Table 3. Overlap of genes belonging to ECM category among SET1 to SET4 datasets.**

| **Datasets** | **Common genes** |
| --- | --- |
| SET1, SET2, SET4 | COL5A1  TIMP3 |
| SET1, SET2 | TGFBR3  MMP19  EFEMP2  APOE |
| SET1, SET3 | TGM2 |
| SET1, SET4 | ADAMTS4  SEMA3C  TRF  TGFBI |

**Supplementary Table 4.** **Genes belonging to “Extracellular matrix” GO category upregulated in DS fetal hearts.** Upregulated genes from Conti et al. 2007, analyzed by Webgestalt software, are enriched in 62 genes belonging to “Extracellular matrix” GO category.

| ID: GO:0031012; Name: extracellular matrix | |
| --- | --- |
| Size = 496; overlap = 62; expect = 11.95; enrichment Ratio = 5.19; FDR < 10e−10 | |
| **User ID** | **Gene Name** |
| ACAN | aggrecan |
| ADAMTS1 | ADAM metallopeptidase with thrombospondin type 1 motif 1 |
| ADAMTS3 | ADAM metallopeptidase with thrombospondin type 1 motif 3 |
| ADAMTS5 | ADAM metallopeptidase with thrombospondin type 1 motif 5 |
| AHSG | alpha 2-HS glycoprotein |
| AMBP | alpha-1-microglobulin/bikunin precursor |
| APOA1 | apolipoprotein A1 |
| APOC3 | apolipoprotein C3 |
| ASPN | asporin |
| AZGP1 | alpha-2-glycoprotein 1. zinc-binding |
| BGN | biglycan |
| CASK | calcium/calmodulin dependent serine protein kinase |
| COL13A1 | collagen type XIII alpha 1 chain |
| COL14A1 | collagen type XIV alpha 1 chain |
| COL15A1 | collagen type XV alpha 1 chain |
| COL18A1 | collagen type XVIII alpha 1 chain |
| COL1A1 | collagen type I alpha 1 chain |
| COL1A2 | collagen type I alpha 2 chain |
| COL3A1 | collagen type III alpha 1 chain |
| COL5A1 | collagen type V alpha 1 chain |
| COL5A2 | collagen type V alpha 2 chain |
| COL6A1 | collagen type VI alpha 1 chain |
| COL6A2 | collagen type VI alpha 2 chain |
| COL9A2 | collagen type IX alpha 2 chain |
| COL9A3 | collagen type IX alpha 3 chain |
| DCN | decorin |
| ECM2 | extracellular matrix protein 2 |
| FBLN1 | fibulin 1 |
| FBN1 | fibrillin 1 |
| FGA | fibrinogen alpha chain |
| FGB | fibrinogen beta chain |
| FGFR2 | fibroblast growth factor receptor 2 |
| FLRT2 | fibronectin leucine rich transmembrane protein 2 |
| GLG1 | golgi glycoprotein 1 |
| GPC3 | glypican 3 |
| GPC4 | glypican 4 |
| HAPLN1 | hyaluronan and proteoglycan link protein 1 |
| ITGB1 | integrin. beta 1 |
| ITGB4 | integrin. beta 4 |
| LAMA4 | laminin subunit alpha 4 |
| LAMC3 | laminin subunit gamma 3 |
| LEFTY2 | left-right determination factor 2 |
| LTBP4 | latent transforming growth factor beta binding protein 4 |
| LUM | lumican |
| MATN2 | matrilin 2 |
| MFAP2 | microfibril associated protein 2 |
| MFAP4 | microfibril associated protein 4 |
| MMP11 | matrix metallopeptidase 11 |
| MMP2 | matrix metallopeptidase 2 |
| OGN | osteoglycin |
| OMD | osteomodulin |
| PCOLCE | procollagen C-endopeptidase enhancer |
| PTN | pleiotrophin |
| RELN | reelin |
| SERPINE2 | serpin family E member 2 |
| SOD3 | superoxide dismutase 3 |
| SPON1 | spondin 1 |
| TGM4 | transglutaminase 4 |
| TIMP2 | TIMP metallopeptidase inhibitor 2 |
| TPSAB1 | tryptase alpha/beta 1 |
| TRIL | TLR4 interactor with leucine rich repeats |
| VCAN | versican |
| WNT4 | Wnt family member 4 |
| WNT5B | Wnt family member 5B |
